# Mitochondria as immunometabolic hubs in psoriasis: pathogenic mechanisms and therapeutic prospects

**DOI:** 10.3389/fimmu.2026.1991331

**Published:** 2026-10-23

**Authors:** Fangru Zhao, Yaoxing Guo, Xin Tong, Xiangnan Zhou, Yingdi Jiao, Renjie Han, Hongduo Chen, Ruiqun Qi

**Affiliations:** 1 Department of Dermatology, The Hospital of China Medical University, Shenyang, China; 2 Key Laboratory of Immunodermatology, Ministry of Education, and National Health Commission; National Joint Engineering Research Center for Theranostics of Immunological Skin Diseases, Shenyang, China

**Keywords:** immunometabolism, Mitochondria, mtROS, psoriasis, targeted metabolic therapy

## Abstract

Psoriasis is a chronic, immune-mediated inflammatory dermatosis characterized by a profound dysregulation of immune-epithelial crosstalk. Beyond canonical ATP production, mitochondria function as essential intracellular immunometabolic hubs embedded within the cytokine-driven network of psoriatic lesions. Within this inflammatory niche, keratinocytes and immune cells undergo structural and metabolic adaptations. Aberrant mitochondrial stress byproducts—including mitochondrial reactive oxygen species (mtROS), oxidized mitochondrial DNA (mtDNA), and metabolic intermediates—act as secondary amplifiers that reinforce innate immune sensing and adaptive T cell responses, thereby sustaining a localized inflammatory loop. Here, we propose that targeting mitochondrial pathways provides a novel, stage-dependent therapeutic framework: stabilizing mitochondrial dynamics during acute flare-ups to halt inflammation, disrupting tissue-resident memory T (T_RM_) cell metabolic fitness during the stable phase to avert relapse, and restoring regulatory T (Treg) cell oxidative phosphorylation (OXPHOS) during regression to re-establish peripheral immune tolerance. Transitioning from broad immunosuppression to precision mitochondrial modulation offers a promising paradigm for cell-specific psoriasis interventions.

## Graphical Abstract

**Figure:**
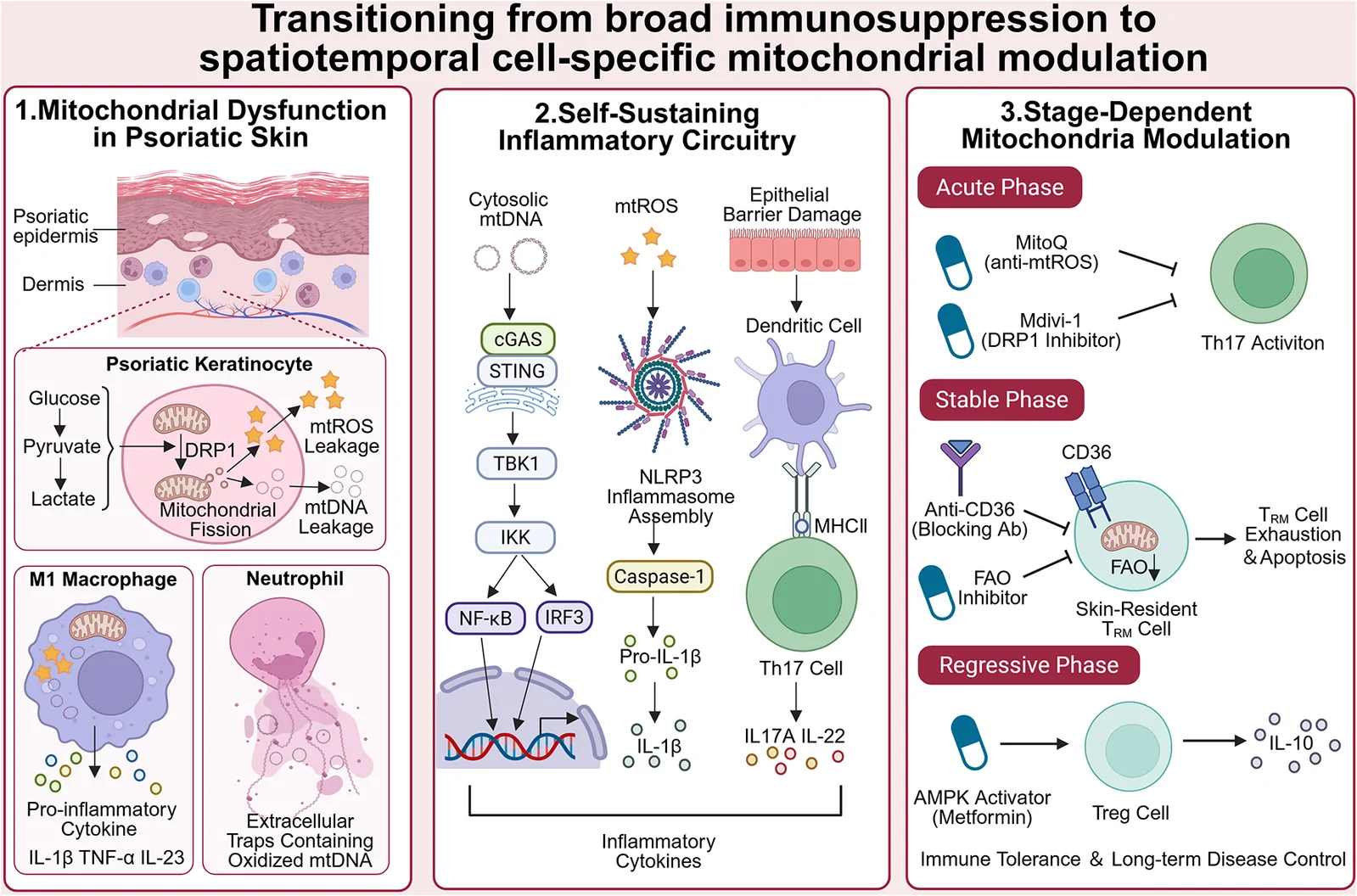
Psoriatic cutaneous inflammation involves prominent mitochondrial dysfunction, which acts as an intracellular metabolic relay and secondary amplifier through the release of endogenous danger signals. Implementing stage-dependent mitochondrial modulation—controlling acute flares, inducing TRM cell exhaustion during remission, and restoring Treg function—presents a promising spatiotemporal strategy to re-establish cutaneous immune tolerance.

## 1 Introduction

Psoriasis is a chronic, immune-mediated inflammatory dermatosis characterized by epidermal hyperproliferation and dermal leukocyte infiltration (1). Although biologics targeting the IL-23/IL-17 axis have revolutionized moderate-to-severe plaque psoriasis management (2), formidable bottlenecks persist, including marked efficacy heterogeneity, high relapse rates, and 10%–30% primary non-response (3). These clinical challenges strongly imply a deep-seated, self-sustaining inflammatory circuitry embedded within the cutaneous microenvironment that evades systemic cytokine blockade alone. Therefore, contemporary research must shift upstream onto the pivotal intracellular and intercellular signaling networks orchestrating skin immunometabolic homeostasis.

In recent years, powered by rapid advancements at the intersection of immunology and metabolism, emerging evidence indicates that perturbations in mitochondrial functional homeostasis serve as critical intracellular amplification nodes operating within persistent inflammatory cascades in immune-mediated diseases (4). Mitochondria have fundamentally transcended their classical definition as mere cellular energy plants; instead, they are recognized as master immunometabolic hubs that dynamically integrate metabolic fluxes, oxidative stress, and inflammatory signaling pathways (5). During ongoing inflammatory responses, mitochondrial metabolic remodeling dynamically calibrates immune cell activation states, supports inflammatory amplification, and influences the maintenance of immune tolerance by fine-tuning reactive oxygen species (ROS) generation, cytosolic leakage of mtDNA, and the availability of tricarboxylic acid cycle intermediates, thereby emerging as a critical structural and metabolic bridge molecularly intertwining metabolic rewiring with chronic tissue inflammation (6).

Consistently, within the psoriatic lesional microenvironment, keratinocytes, innate immune cells, and adaptive immune cells all exhibit prominent mitochondrial structural remodeling, metabolic derangements, and quality control defects (7). This review systematically integrates the latest research breakthroughs at the intersection of psoriasis and immunometabolism to comprehensively dissect the discrete mitochondrial abnormalities across keratinocytes, innate immune cell subsets, and adaptive immune cells, with a particular focus on the pivotal role of mitochondria-mediated intercellular communication networks in driving localized inflammatory amplification. Furthermore, by harmonizing the distinct clinicopathological hallmarks of the acute flare-up, stable, and regressive phases of psoriasis, we introduce a novel, stage-dependent framework for the comprehensive management of targeted mitochondrial metabolism: stabilizing mitochondrial dynamics during the acute phase to halt the inflammatory cascade; disrupting the metabolic fitness of T_RM_ cells during the stable phase to extend clinical remission; and restoring regulatory T_reg_ cell mitochondrial function during the regressive phase to re-establish peripheral immune tolerance. In summary, this conceptual framework provides essential theoretical scaffolding and transitional directions to prompt a paradigm shift in psoriasis therapeutics—from conventional, broad-spectrum immunosuppression toward precision, dual mitochondrial metabolic-immunological modulation.

## 2 Fundamental functions of mitochondria in immunomodulation

Mitochondria are the bioenergetic cornerstones of eukaryotic cells, integrating metabolic fluxes to maintain homeostatic requirements (8). Within the matrix, the tricarboxylic acid (TCA) cycle oxidizes carbohydrates, fatty acids, and amino acids to generate reducing equivalents (NADH FADH_2_), which fuel OXPHOS across the inner membrane for ATP synthesis (9). Concurrently, mitochondria actively participate in a myriad of biosynthetic processes, such as the synthesis of lipids, nucleotides, heme, and iron-sulfur clusters, thereby serving as a vital nexus in the cellular metabolic network (10). Beyond these canonical bioenergetic functions, accumulating evidence indicates that mitochondria critically modulate inflammatory signaling cascades (11)and cellular functional adaptations by dynamically regulating redox homeostasis, intermediate metabolite abundance, and retrograde signaling molecules (12). Consequently, perturbations in mitochondrial homeostasis invariably precipitate metabolic derangements and immunological aberrations (13),firmly establishing these organelles as an essential immunometabolic crossroads intertwining metabolic rewiring with chronic inflammation (14).

During early inflammatory initiation, cells undergo profound metabolic reprogramming. The primordial hallmark is a switch from efficient OXPHOS to aerobic glycolysis (15), coupled with a broken, non-continuous TCA cycle typically caused by diminished succinate dehydrogenase (SDH) activity (16). These accumulated metabolites persistently drive and sustain the inflammatory activation state of immune cells at the metabolic level by modulating transcriptional regulation, epigenetic modifications, and redox equilibrium (17). Concurrently, mitochondrial lipid fluxes rewire from catabolic fatty acid oxidation (FAO) to anabolic *de novo* fatty acid synthesis, a transition that triggers an mtROS burst and reinforces pro-inflammatory phenotypes (18, 19).

As inflammation progresses, sustained metabolic stress precipitates pathological alterations in mitochondrial ultrastructure and morphology, marked by the loss of membrane potential and matrix swelling. Mitochondria actively maintain their functional and structural integrity through a continuous orchestration of fusion and fission events (20).When this dynamic equilibrium is disrupted, excessive fission renders mitochondria highly vulnerable to membrane permeabilization, electron leakage, and an explosive mtROS burst (21). Owing to their bacterial endosymbiotic ancestry, mitochondria harbor immunogenic circular DNA that remains strictly segregated by the double-membrane system under physiological conditions (22). Under severe cellular stress, compromised membrane gating permits the translocation of oxidized mtDNA into the cytosol, where it directly binds the intracellular nucleic-acid sensor cyclic GMP–AMP synthase (cGAS)s (23).This interaction engages stimulator of interferon genes (STING) to activate downstream TBK1, IRF3, and canonical NF-κB cascades, driving the transcription of type I interferons and supportive pro-inflammatory cytokines. Consequently, damaged mitochondria act as autonomous sources of endogenous danger signals, translating organelle stress into sustained tissue inflammation (24).

Persistent structural damage to mitochondria inevitably culminates in mitochondrial outer membrane permeabilization (MOMP), precipitating the massive efflux of pro-apoptotic factors, such as cytochrome c, from the mitochondrial matrix into the cytosol. Upon translocating into the cytosol, cytochrome c binds to apoptotic protease activating factor-1 (Apaf-1) to orchestrate the assembly of the apoptosome, which subsequently triggers the downstream Caspase-9/3 signaling cascade to execute the cell apoptosis program (25).This mitochondrion-centered programmed cell death not only accelerates the structural dissolution of the localized epidermal barrier but also generates extracellular debris during cellular disintegration. These cellular fragments subsequently function as secondary danger signals, progressively exacerbating the pro-inflammatory cascades of adjacent innate immune cells (26).

Throughout these cascades, mitophagy functions as an indispensable negative feedback brake system (27). Conversely, compromised mitophagy initiates a deleterious feed-forward loop encompassing unmitigated organelle injury, persistent DAMP release, and self-sustaining tissue inflammation (28).

## 3 Mitochondrial dysfunction in psoriatic keratinocytes

Accumulating evidence indicates that psoriasis is far from a simplistic, linear cascade driven exclusively by immune cells acting upon downstream keratinocytes; instead, it is characterized by the formation of a localized, intra-epidermal inflammatory niche co-sustained by reciprocal interactions between keratinocytes and immune signaling networks (29, 30). Recent single-cell and spatial transcriptomic investigations have unveiled pronounced spatial compartmentalization and functional heterogeneity within the psoriatic epidermis. Notably, within an IL-36-dependent microenvironment, keratinocytes can autonomously amplify IL-17A and TNF-α related inflammatory transcriptional programs, thereby establishing a self-sustaining, feed-forward loop of inflammatory amplification within the epidermal architecture (31).These pioneering findings underscore that keratinocytes are not merely passive targets of immune-derived cytokines, but active orchestrators of localized inflammatory chronicity. In this context, mitochondrial metabolic remodeling, organelle stress signaling, and regulated cell death pathways provide the requisite bioenergetic adaptation and pathophysiological conduits that sustain this pathogenic epithelial phenotype.

Against this backdrop, core pro-inflammatory cytokines in psoriatic lesions directly initiate mitochondrial structural and respiratory impairment in keratinocytes. Substantiated by both *in vitro* assays and imiquimod (IMQ)-induced psoriasiform murine models, the synergistic stimulation of IL-17A, IL-22, and TNF-α provokes mitochondrial network fragmentation, respiratory chain collapse, and an aberrant burst of mtROS, accompanied by pathological cristae dissolution and matrix swelling (32). These findings demonstrate that the cytokine-rich microenvironment autonomously imposes severe organelle stress on keratinocytes, operating independently of direct immune-cell contact.

In response to this persistent organelle stress, psoriatic keratinocytes undergo comprehensive remodeling of metabolic fluxes and the intermediate metabolite spectrum to satisfy the bioenergetic and biosynthetic demands of epidermal hyperproliferation (32). Diverging from the simplistic paradigm of an exclusive shift from OXPHOS to glycolysis, keratinocytes exhibit marked metabolic plasticity characterized by spatial compartmentalization across the psoriatic epidermal strata (33). In the suprabasal layers, the CD147–GLUT1 axis accelerates glucose uptake to drive aerobic glycolysis; this axis subsequently orchestrates a metabolic-epigenetic coupling mechanism revolving around the α-KG–H3K9me3–*BBOX1* axis, which facilitates keratinocyte hyperproliferation and sustains psoriasiform inflammation (33). Conversely, basal keratinocytes, operating under localized glucose competition, display a distinct *COX7B*-mediated upregulation of OXPHOS coupled with a calibrated ROS burst to preserve their high proliferative potential (34). Consequently, metabolic reprogramming in psoriatic keratinocytes represents a dynamic, multi-layered process of mitochondrial metabolic adaptation rather than a unidirectional shutdown of oxidative respiration.

Building upon this spatial metabolic shift, persistent hyper-glycolysis in the suprabasal epidermis orchestrates epigenetic aberrations that disrupt mitochondrial integrity. Mechanistically, heightened glycolytic flux prompts the abnormal expression of histone deacetylase 3 (*HDAC3*), which stimulates excessive mtROS generation, promotes mitochondrial outer membrane permeabilization, and drives the extrusion of oxidized mtDNA into the cytosol (35). Rather than initiating *de novo* inflammation, cytosolic mtDNA engages the cGAS–STING axis as a secondary amplifier to fuel pro-inflammatory cytokine release and epidermal hyperplasia. Downstream of STING, this signaling cascades into IκBα phosphorylation and the nuclear translocation of canonical NF-κB (p65), which synergizes with IRF3 to drive the transcription of key epidermal cytokines and chemokines (36). Demonstrating the translational relevance of this circuit, targeted pharmacological inhibition of *HDAC3* effectively blunts cytosolic mtDNA accumulation and ameliorates IMQ-induced psoriasiform lesions in mice (35), while the small-molecule STING antagonist H-151 yields comparable therapeutic protection (36).

Beyond driving cytosolic nucleic-acid sensing, excessive mtROS accumulation and respiratory electron leakage trigger extensive membrane lipid peroxidation in keratinocytes. Sustained mitochondrial oxidative stress directly couples to ferroptosis—an iron-dependent, non-apoptotic regulated cell death mode governed by perturbed organelle lipid metabolism and exhaustion of the glutathione–glutathione peroxidase 4 (GPX4) antioxidant axis (37). Substantiated by cultured myeloid cells and IMQ-induced murine models, impaired mitochondrial redox buffering synergizes with labile iron overload to accelerate the toxic peroxidation of polyunsaturated fatty acids, leading to plasma membrane rupture, epithelial barrier collapse, and the extrusion of pro-inflammatory cellular debris (38). From a pathophysiological and therapeutic perspective, this mitochondrion-dependent ferroptotic cascade not only acts as a secondary amplifier of localized cutaneous chronicity by restimulating dermal immune subsets, but also highlights targeted inhibition of ferroptosis—such as via topical radical-trapping antioxidants or iron chelators—as a viable approach to restore epidermal integrity and blunt psoriatic inflammation (38).

Concurrently, defects in the mitochondrial quality control machinery exacerbate localized epithelial inflammation. Under physiological conditions, mitophagy selectively eliminates damaged, ROS-generating mitochondria to maintain epithelial homeostasis (39, 40). In IMQ-induced murine models, reduced adenosine monophosphate-activated protein kinase (AMPK) activity impairs both ULK1/Atg7-dependent autophagic initiation and PINK1/Parkin-mediated organelle tagging, leading to a marked blockade of mitophagic clearance (41). Consequently, the persistence of dysfunctional mitochondria sustains elevated mtROS production and retrograde danger signaling, which impairs normal keratinocyte differentiation and promotes chronic cytokine secretion (42, 43). Pharmacological activation of AMPK or restoration of mitophagic flux efficiently restores organelle clearance and attenuates psoriasiform skin inflammation in preclinical studies (41).

In summary, mitochondrial abnormalities in psoriatic keratinocytes encompass a complex network of cytokine-induced structural damage, regional metabolic adaptations, secondary nucleic-acid sensing, ferroptotic barrier collapse, and defective quality control (**Figure 1**). Rather than acting merely as downstream targets of immune signals, keratinocytes utilize these organelle-driven pathways to amplify localized inflammation, reinforcing a persistent pathogenic circuit with infiltrating cutaneous leukocytes.

**Figure 1:**
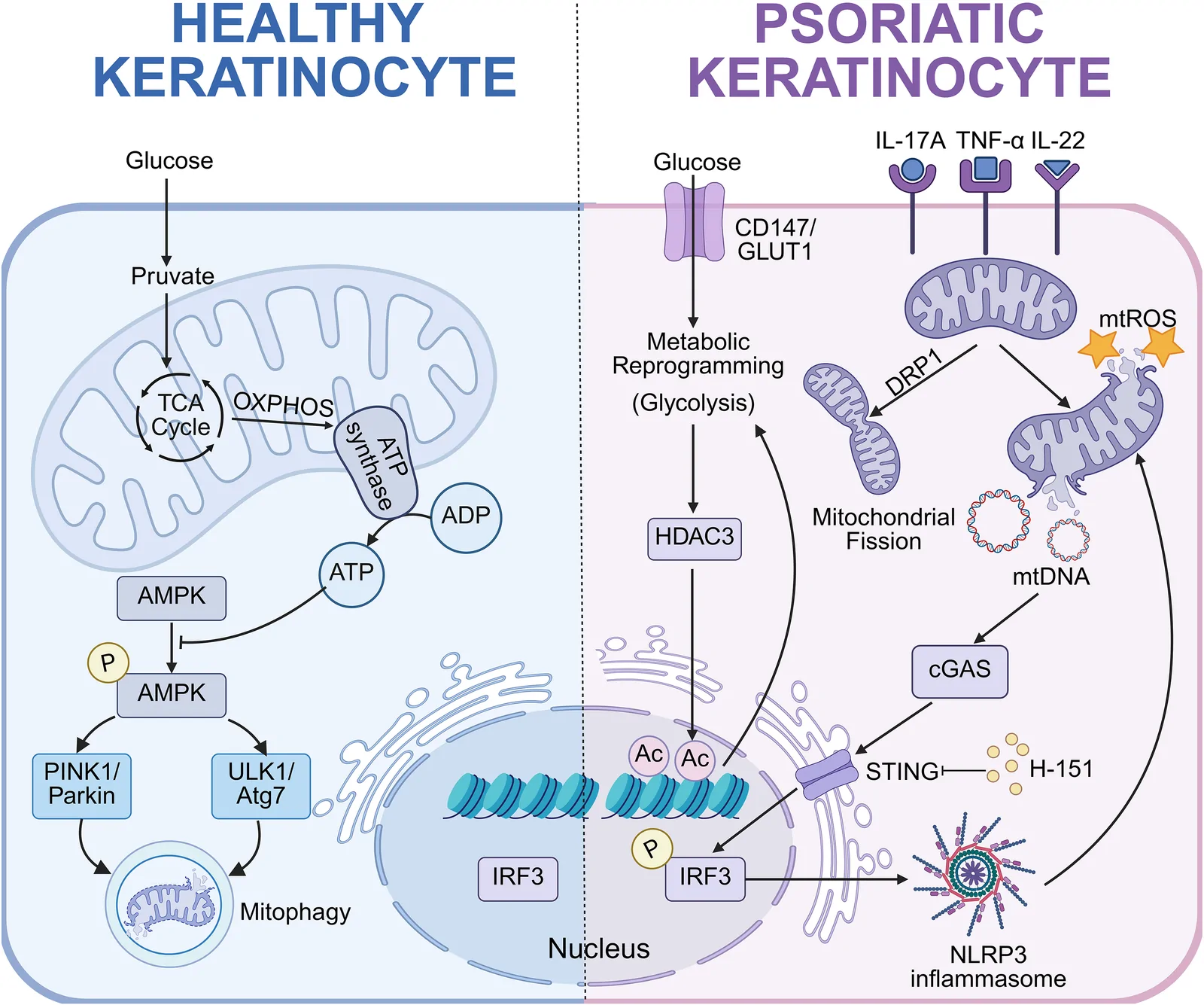
Mitochondrial dysfunction and inflammatory signaling regulatory networks in psoriatic keratinocytes. Left panel (Healthy State): Under physiological homeostasis, active AMPK signaling coordinates the initiation of both PINK1/Parkin- and ULK1/Atg7-mediated mitophagy cascades, which selectively clear damaged mitochondrial fragments to preserve baseline cellular metabolism and redox equilibrium. Right panel (Psoriatic State): Microenvironmental pro-inflammatory cytokines trigger extensive mitochondrial structural disintegration and metabolic rewiring, characterized by three interconnected pathogenic modules: (1) Metabolic reprogramming: The CD147–GLUT1 signaling axis accelerates glucose uptake to fuel hyper-glycolysis, while aberrFOXPant upregulation of *HDAC3* provides epigenetic sustenance to sustain enhanced glycolytic fluxes and hyperproliferative phenotypes. (2) Dynamics and quality control defects: Upregulation of DRP1 drives pathological mitochondrial fission and network fragmentation, while synchronized suppression of AMPK signaling blocks mitophagy clearance, culminating in the deleterious accumulation of compromised organelles. (3) Innate immune sensing: Structural architecture collapse provokes an explosive burst of mtROS and the anomalous extrusion of cell-free mtDNA into the cytosol. Cytosolic mtDNA acts as a prototypical DAMP to ignite the cGAS–STING–IRF3 axis, cooperatively synergizing with mtROS to hyperactivate the NLRP3 inflammasome, thereby amplifying localized epidermal inflammation. This pathogenic cascade can be successfully arrested via pharmacological intervention with the small-molecule STING antagonist H-151.

## 4 Mitochondrial regulation in innate immune cells during psoriasis

Mitochondria-mediated inflammatory responses are strictly non-confined to the epidermal layer; indeed, innate immune cell subsets embedded within the superficial dermis—including macrophages, dendritic cells, and neutrophils—exert pivotal regulatory roles within the psoriatic lesional microenvironment (44, 45). Coupled with environmental cytokine cues, mitochondrial metabolic remodeling, dynamic alterations, and oxidative stress cascades act as critical intracellular facilitators supporting the pro-inflammatory polarization and effector functions of innate immune cells. Rather than operating as solitary disease initiators, these mitochondrial adjustments function as adaptive metabolic relays that sustain prolonged myeloid cell activation, thereby reinforcing the cascade amplification of tissue inflammation (4).

During inflammatory activation, macrophages switch from oxidative phosphorylation toward aerobic glycolysis (46–48), while co-stimulatory signals (such as 4-1BBL) induce *de novo* lipogenesis to sustain pro-inflammatory cytokine production (49). In psoriatic skin, this baseline state is further influenced by local lipid availability. Analyses of patient biopsies and animal models show that infiltrating macrophages upregulate fatty acid-binding protein 5 (FABP5) to import extracellular saturated fatty acids, which lowers the threshold for pro-inflammatory activation and enhances IL-1β expression (50, 51).

Concurrently, studies in animal models and cultured cells demonstrate that macrophage activation coincides with marked mitochondrial structural alterations: aberrant activation of the GDAP1L1/DRP1 axis accelerates mitochondrial fission and mtROS generation, thereby augmenting the secretion of TNF-α, IL-6, and IL-1β (52). Although FABP5-mediated lipid uptake and GDAP1L1-driven mitochondrial fission were identified independently, whether accumulated intracellular saturated fatty acids act as a lipotoxic trigger that directly promotes GDAP1L1 activation and subsequent fragmentation remains to be experimentally validated.

Beyond driving M1 polarization, whether unresolved mitochondrial damage actively blocks the phenotypic transition toward an anti-inflammatory M2 state remains an open question. Persistent organelle dysfunction and sustained cGAS–STING activation may lock macrophages into a non-resolving pro-inflammatory state, thereby impairing tissue repair and fueling chronicity. Elucidating the metabolic barriers that impede this phenotypic switch could unveil targeted strategies to restore resolution in psoriatic lesions.

Serving as a critical immunological bridge interconnecting innate and adaptive immunity, the metabolic programs of dendritic cells (DCs) are intricately coupled with their pro-inflammatory biosynthetic outputs. Upon immunological activation, DCs undergo a metabolic shift from a resting state sustained by OXPHOS toward a highly active, pro-inflammatory glycolytic program. This bioenergetic rewiring provides the metabolic plasticity and energetic reservoir required for efficient antigen presentation, thereby fulfilling the high biosynthetic demands for producing key cytokines, including IL-12, IL-23, and type I interferons. Crucially, these soluble mediators constitute decisive upstream drivers of the pathogenic Th17-cell response in psoriasis (2, 30, 53). Furthermore, mitochondrion-derived TCA intermediates and mtROS function as retrograde signals, orchestrating the pro-inflammatory commitment of DCs via epigenetic modifications or transcription factor activation (54).

In addition to conventional DCs, plasmacytoid dendritic cells (pDCs) infiltrate early psoriatic skin and produce high levels of type I interferons (primarily IFN-α) upon sensing self-DNA complexes (55). Unlike conventional myeloid cells that rely heavily on glycolysis, pDCs require functional mitochondrial OXPHOS and FAO to sustain their baseline bioenergetics. By maintaining cellular ATP pools and restricting excessive oxidative stress, intact respiratory chain activity effectively licenses endosomal TLR7/9 trafficking and subsequent IRF7-driven IFN-α production (56). Consequently, intact mitochondrial respiration serves as a metabolic requirement for pDC activation during the initial phase of psoriatic inflammation.

Although our mechanistic understanding of mitochondrial biology in myeloid cells has progressed, systematic investigations into DCs within psoriatic lesions remain notably scarce. This gap is primarily bottlenecked by their low anatomical abundance and extreme cellular heterogeneity, which render traditional bulk-sequencing and *in vitro* assays incapable of capturing subtle, *in situ* metabolic flux dynamics. Consequently, prevailing studies often treat global DCs as a single entity, obscuring the discrete, heterogeneous metabolic reprogramming that characterizes specific functional subclusters, such as inflammatory DCs and cDC2s (57, 58). Looking ahead, leveraging single-cell spatial metabolomics alongside intravital imaging to chart the precise *in situ* metabolic blueprints of discrete DC subclusters is imperative (59). Dissecting whether individual subsets differentially dictate core cytokine production (e.g. IL-23) by selectively utilizing distinct mitochondrial substrates will be instrumental in decoding the psoriatic immune microenvironment.

Neutrophil accumulation is a hallmark histopathological feature across clinical variants of psoriasis, manifesting as Munro’s microabscesses within the stratum corneum in plaque psoriasis and Kogoj’s spongiform pustules in generalized pustular psoriasis (60–62). Beyond their conventional phagocytic functions, neutrophils isolated from psoriatic patients and imiquimod-induced mouse models display marked mitochondrial quality control abnormalities, predominantly driven by pathological DRP1-mediated mitochondrial fission (61, 63). This hyper-fission disrupts mitochondrial cristae architecture and uncouples the electron transport chain, precipitating an mtROS surge and loss of mitochondrial membrane potential (63, 64). The accumulated mtROS functions as an intrinsic danger signal that triggers NLRP3 inflammasome assembly, which drives caspase-1 activation and licenses the extrusion of neutrophil extracellular traps (NETs) (65, 66). Notably, the abundance of circulating and lesional NETs correlates positively with clinical disease severity (PASI scores), directly implicating mitochondrion-dependent NETosis in driving local tissue inflammation (65).

A defining characteristic of psoriatic NETosis is the biological fate of extruded mtDNA (67, 68). Neutrophil-derived mtDNA undergoes extensive oxidative modification (8-OHdG) during the respiratory burst, rendering it intrinsically resistant to rapid degradation by extracellular nucleases (67). In psoriatic plaques, this oxidized mtDNA readily binds to cationic antimicrobial peptides, primarily LL-37, or becomes packaged into neutrophil-derived extracellular vesicles (67, 69). These complexes overcome the electrostatic barriers of target cell membranes, facilitating their endocytic uptake by resident myeloid dendritic cells and macrophages (69). Inside the recipient cells, the internalized mtDNA stimulates pattern recognition receptors to initiate the transcriptional upregulation of type I interferons, IL-23, and downstream neutrophil-attracting chemokines, such as CXCL1, CXCL2, and CXCL8 (69, 70). This creates a pathogenic feed-forward loop in which mitochondrial breakdown products perpetually recruit subsequent waves of neutrophils into the lesion (71).

In summary, mitochondria transcend their classical roles as mere energetic powerhouses for innate immune cells to function as the central homeostatic rheostat dictating both their pro-inflammatory phenotypes and activation kinetics. Although discrete variations exist in the precise mitochondrial manifestations among macrophages, dendritic cells, and neutrophils, these three major innate immune subsets share convergent pathological features characterized by mitochondrial metabolic reprogramming, dynamic fission-fusion imbalances, and quality control deficits. Operating in unison, these mitochondrial adaptations synergistically support the pathological activation of innate immunity, thereby serving as an amplification relay that reinforces and perpetuates the localized immunological aberrations in psoriasis (**Figure 2**).

**Figure 2:**
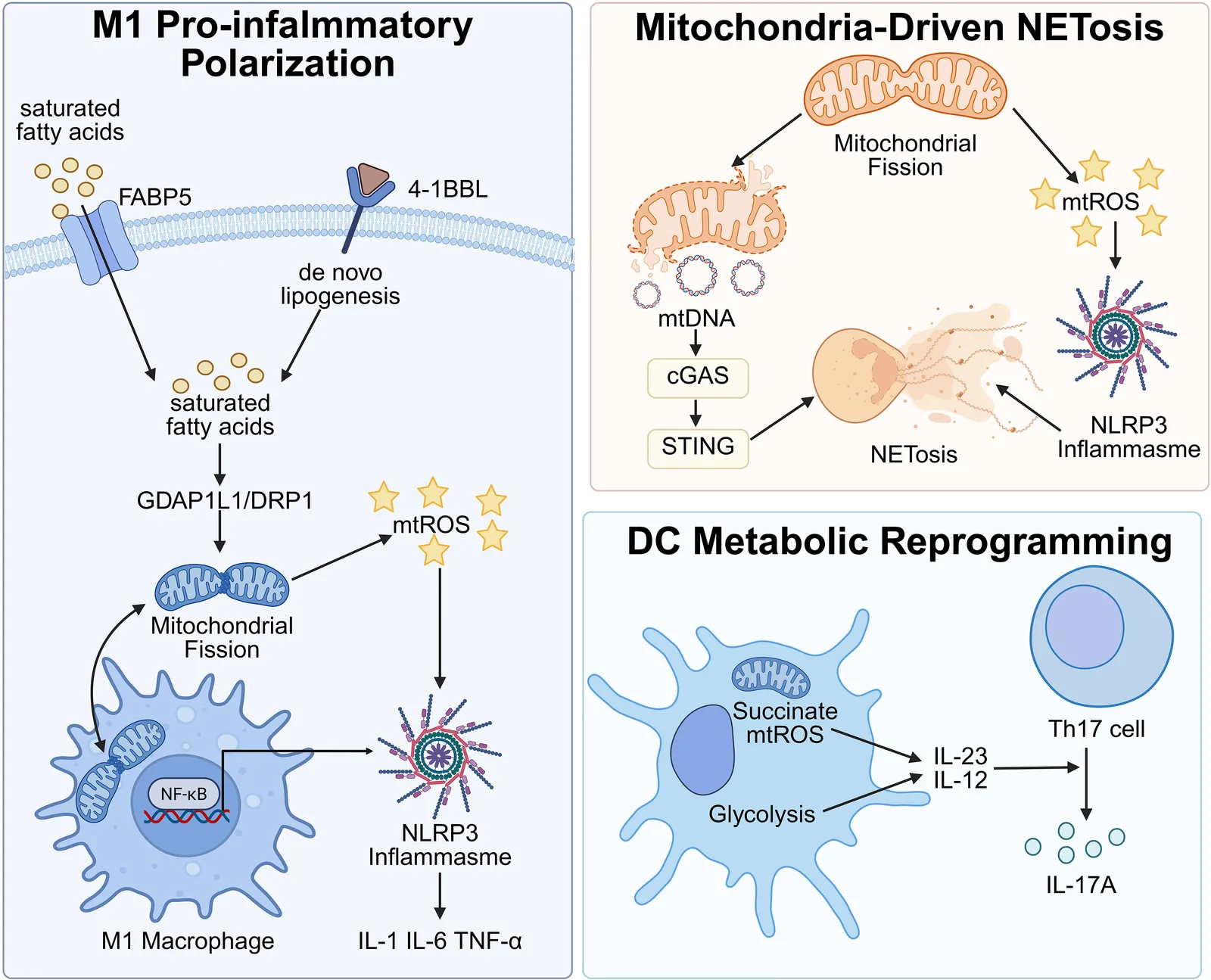
Mitochondrial regulation in the activation of innate immune cells in psoriasis. Macrophages: The 4-1BBL axis drives *de novo* lipogenesis, while FABP5 mediates extracellular saturated fatty acid uptake. This joint metabolic stress activates the GDAP1L1/DRP1 axis to accelerate mitochondrial fission, locking the pro-inflammatory M1 phenotype and triggering an mtROS burst that synergizes with NF-κB and NLRP3 pathways to promote IL-1β, IL-6, and TNF-α release. Neutrophils: Excessive mitochondrial fission disrupts electron transport chain efficiency, leading to mtROS accumulation. Elevated mtROS activates the NLRP3 inflammasome and induces NETosis; the released cell-free mtDNA acts as a potent ligand to chronically activate the cGAS–STING pathway, amplifying chronic inflammation. Dendritic Cells: Primed DCs undergo a metabolic switch from OXPHOS to glycolysis, accompanied by the accumulation of intracellular mtROS and TCA cycle intermediates. This rewiring regulates the expression of core upstream cytokines IL-23 and IL-12, thereby driving the pathogenic Th17-cell response *in situ*.

## 5 Mitochondrial metabolic characteristics of adaptive immune cells in psoriasis

Adaptive immune cells, particularly T cells, serve as the primary drivers of the initiation, maintenance, and relapse of psoriasis. The T-cell immune network, centered around the IL-23/IL-17 axis, is widely recognized as the most critical pathogenic pathway in this disease; within this network, helper T 17 (Th17) cells, γδT17 cells, and T_RM_ cooperatively drive the development of cutaneous lesions, whereas the functional impairment of Tregs further compromises immunological tolerance (72).

Accumulating evidence demonstrates that T-cell effector functions are tightly coupled to their metabolic programs. Distinct T-cell subsets rely on tailored baseline mitochondrial activities that govern their activation thresholds (73), tissue survival, and cytokine biosynthetic outputs (12, 13). Grounded in this immunometabolic framework, targeting T-cell mitochondrial metabolic adaptations has demonstrated notable translational potential in immune-mediated disorders (74).

As the major producers of IL-17A in psoriatic skin, the clonal expansion and activation of Th17 and γδT17 cells correlate with clinical disease severity (30). Upon immune activation, the effector program of Th17 and γδT17 cells relies on a coordinated metabolic rewiring that couples elevated aerobic glycolysis with functional mitochondrial respiration (75).Rather than a simple shutoff of mitochondrial respiration, activated Th17 and γδT17 cells display marked metabolic plasticity: they accelerate aerobic glycolysis to fuel rapid clonal proliferation while concurrently preserving functional mitochondrial OXPHOS to sustain biosynthetic fluxes and retrograde signaling.

In the inflamed cutaneous milieu, this metabolic configuration is reinforced by DRP1-mediated mitochondrial fission, which supports metabolic branching and calibrated mtROS generation without triggering cell death (76). In murine models of psoriasiform inflammation, targeted inhibition of mitochondrial translation in Vγ4^+^ γδT cells blunts their IL-17A secretion and attenuates epidermal lesions (77, 78). These findings demonstrate that functional mitochondrial respiration acts as a metabolic requirement and signaling platform that sustains the pathogenic effector functions of IL-17-producing T cells.

In addition to glucose metabolism, lipogenic remodeling plays a critical role in establishing the pathogenic phenotypes of Th17 and γδT17 cells. The polarization of canonical pro-inflammatory T cells hinges heavily upon ACC1-mediated *de novo* lipogenesis; the endogenous lipid fractions synthesized through this enzymatic pathway function as physiological ligands that bind to and activate RORγt, thereby driving pathogenic Th17 commitment (79). Consistently, pharmacological blockade or genetic disruption of the *ACC1* pathway markedly dampens IL-17A secretion by γδT17 cells and substantially attenuates epidermal hyperplasia within psoriasiform mouse models (79, 80). These findings indicate that *de novo* fatty acid synthesis serves as an important metabolic supporter that facilitates the sustained pro-inflammatory activation and lineage maintenance of these pathogenic T cells.

Psoriasis is characterized by a pronounced propensity for clinical recurrence, with tissue-resident memory T_RM_ cells widely recognized as the crucial cellular drivers orchestrating this localized relaps (81, 82). Persisting long-term in resolved skin lesions, T_RM_ cells rapidly produce IL-17A and IFN-γ upon secondary triggers. Emerging immunometabolic evidence underscores that T_RM_ cells typically rely upon mitochondrial OXPHOS integrated with FAO to fuel their long-term survival; this tailored metabolic signature guarantees their energetic fitness and persistence within the competitive, inflamed tissue niches (83, 84). Consistent with this paradigm, under psoriatic inflammatory conditions, optimizing immune cell mitochondrial functionality successfully blunts the pathogenic IL-17/STAT3 signaling cascade within CD8^+^ T_RM_ cells by minimizing mtROS accumulation, thereby substantially mitigating psoriasiform lesions (85). Given that T_RM_ persistence is metabolically tied to lipid catabolism and mitochondrial homeostasis, enhanced mitochondrial adaptation within localized T_RM_ clusters likely governs the kinetics of psoriatic relapse.

Treg cells exert a critical gatekeeping role in maintaining peripheral immunological tolerance; however, patients with psoriasis consistently exhibit multifaceted abnormalities in both Treg abundance and suppressive capacity (72). In contrast to the glycolytic bias of Th17 cells, Tregs rely on mitochondrial OXPHOS and FAO to maintain lineage stability and immunosuppressive activity (86, 87). Consequently, impaired mitochondrial respiration or diminished OXPHOS efficiency compromises *FOXP3* expression, predisposing Tregs to lose suppressive function or convert toward a pro-inflammatory phenotype.

Accumulating evidence has substantiated that rejuvenating mitochondrial functionality or fueling fatty acid catabolism successfully restores the suppressive apparatus of Tregs, whereas the targeted dampening of mitochondrial metabolism severely cripples their immunoregulatory functions (88–90). Although direct investigations delineating Treg mitochondrial metabolism specifically within psoriatic tissue remain emerging, integrating evidence of impaired Treg suppressive capacity in psoriasis (72) with their strict energetic reliance on mitochondrial respiration (91) suggests that mitochondrial quality control defects and compromised OXPHOS likely impair Treg suppressive stability, thereby weakening the regulatory checkpoint that normally restrains pathogenic effector T cells.

In conclusion, the protracted progression and persistent clinical recurrence of the adaptive immune response in psoriasis are intrinsically governed by the discrete, stage-dependent mitochondrial metabolic states adopted by individual T-cell subsets, thereby unlocking viable avenues for stage-specific, targeted metabolic interventions. During the acute exacerbation phase, targeting the mitochondrial metabolic dependencies of Th17 and γδT17 cells—such as by attenuating aberrant DRP1-mediated fission or inhibiting mitochondrial protein synthesis—can suppress excessive IL-17A release and limit plaque expansion.

During stable or resolved phases, therapeutic strategies should focus on eliminating or disarming pathogenic T_RM_ populations in the skin. Locally inhibiting fatty acid uptake or oxidation could selectively compromise the metabolic fitness of resident T_RM_ cells without broadly impairing systemic tissue homeostasis, thereby delaying or preventing clinical flares. Throughout the overarching course of the disease, rejuvenating Treg-mediated immunological tolerance remains the key checkpoint for reconstructing immune homeostasis; specifically, by boosting mitochondrial biogenesis or refining mitochondrial quality control circuits, it holds immense promise to reverse the deleterious pro-inflammatory phenotypic conversion of Tregs and restore their OXPHOS- and FAO-dependent immunosuppressive function. This stage-targeted approach provides a conceptual framework for integrating immunometabolic modulation across the clinical trajectory of psoriasis (**Figure 3**).

**Figure 3:**
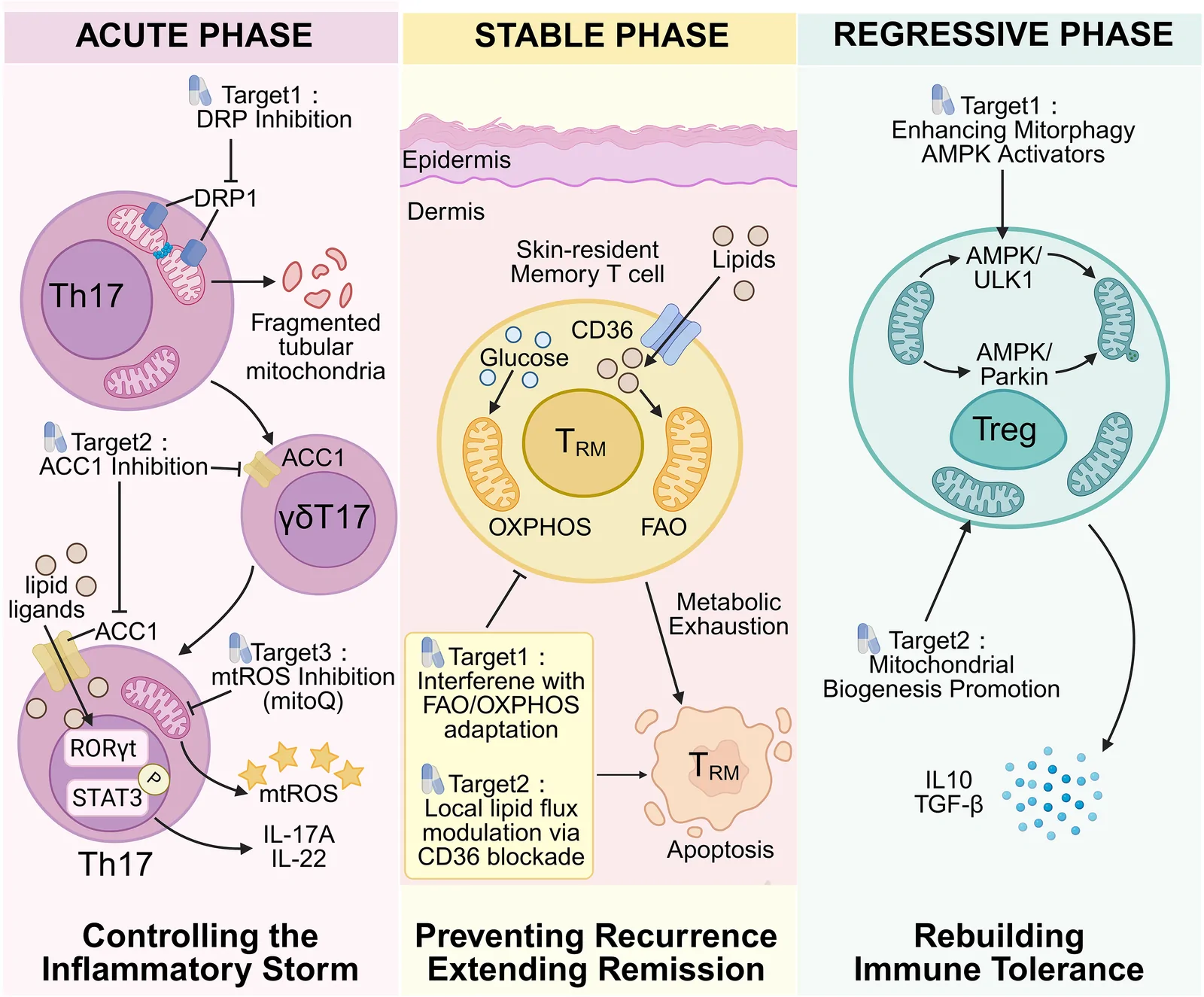
Stage-specific, mitochondrial-targeted therapeutic strategies for adaptive immunity in psoriasis. Acute Exacerbation Phase: Activated Th17 and γδT17 cells shift to glycolysis with DRP1-mediated mitochondrial fragmentation. Fragmented mitochondria cause an mtROS burst to activate the STAT3 pathway, driving IL-17A and IL-22 release. Concurrently, ACC1-mediated *de novo* lipogenesis provides lipid ligands to activate RORγt and lock the pathogenic Th17 phenotype. Interventions target DRP1 (Target 1), ACC1 (Target 2), and mtROS via MitoQ (Target 3) to suppress the inflammation. Stable Phase: Dermal T_RM_ cells depend on CD36-mediated lipid uptake and mitochondrial FAO/OXPHOS for long-term survival. Disruption of FAO/OXPHOS (Target 1) and CD36-mediated lipid flux (Target 2) induces metabolic exhaustion and apoptosis in T_RM_ cells, extending clinical remission. Regressive Phase: Tregs require OXPHOS and FAO for immunosuppression, which is impaired in psoriasis by mitochondrial quality control deficits. Activating AMPK to enhance mitophagy (Target 1) and promoting mitochondrial biogenesis (Target 2) reverses Treg pathogenic conversion and restores peripheral tolerance.

## 6 Mitochondrial-mediated communication networks between keratinocytes and immune cells

The chronicity of psoriatic plaques relies on reciprocal, feed-forward crosstalk between epidermal keratinocytes and dermal immune cells (92, 93). Within active lesions, both compartments undergo concurrent mitochondrial remodeling (93). Rather than functioning solely as cell-autonomous stress events, damaged mitochondria generate diffusible metabolic intermediates, cell-free mitochondrial nucleic acids, and mitochondria-derived extracellular vesicles (MDEVs) that traverse the dermo-epidermal junction to reinforce localized inflammatory circuits.

A primary mode of this metabolic crosstalk is the paracrine signaling driven by intermediate metabolites. In active psoriatic lesions, spatial metabolomics has revealed marked intralesional accumulations of succinate and lactate, which correlate spatially with localized IL-17 expression (94). Extracellular succinate functions as an endogenous ligand that selectively engages the cell-surface receptor SUCNR1 (GPR91) expressed on dermal dendritic cells and macrophages (95). Ligation of SUCNR1 stabilizes intracellular HIF-1α, thereby sustaining the continuous transcription of pro-inflammatory cytokines such as IL-1β—a decisive co-stimulatory factor required for IL-17 production by local T cells (94, 95). Consequently, metabolic efflux from hyper-glycolytic, stressed keratinocytes establishes a localized chemical gradient that primes dermal antigen-presenting cells to sustain the IL-23/IL-17 axis.

In addition to soluble metabolites, cell-free mtDNA released from damaged or ferroptotic keratinocytes serves as a potent paracrine DAMP across the dermo-epidermal junction (96, 97). In psoriatic plaques, extruded oxidized mtDNA is intrinsically resistant to rapid nuclease degradation, and its stability is further enhanced through electrostatic complexation with keratinocyte-derived antimicrobial peptides, particularly LL-37 (67, 68). These cationic complexes facilitate the endocytic uptake of mitochondrial DNA across the plasma membranes of dermal dendritic cells and macrophages (69). Once delivered into the recipient cytosol, this internalized mtDNA activates intracellular nucleic-acid sensing cascades to stimulate sustained IL-23 and type I interferon transcription, thereby establishing an inter-compartmental feed-forward loop that reinforces chronic T-cell activation and epidermal hyperplasia (97).

Furthermore, extracellular vesicles containing mitochondrial components (mitochondria-derived extracellular vesicles, or MDEVs) represent a specialized vehicle for inter-organelle and intercellular signaling (97, 98). Proteomic and molecular analyses of extracellular vesicles isolated from psoriatic plaques and patient sera reveal marked enrichment of mitochondrial inner-membrane proteins, matrix enzymes, and fragmented mtDNA segments (99, 100). When released by stressed or metabolically perturbed keratinocytes into the superficial dermis, these MDEVs deliver oxidized lipids, carbonylated proteins, and intact mitochondrial complexes directly to recipient myeloid subsets (100, 101). Following internalization, this organelle-derived cargo fuels intracellular oxidative stress and primes dendritic cells and macrophages toward a pro-inflammatory state (101). Consequently, disrupting this vesicle-mediated circuitry—either by limiting MDEV biogenesis in stressed keratinocytes or blocking their uptake by dermal immune cells—emerges as an actionable therapeutic entry point to intercept localized inflammatory propagation (101, 102).

## 7 Mitochondrial-targeted therapeutic strategies for psoriasis

In recent years, strategies aimed at mitigating mitochondrial stress signaling have garnered growing interest in preclinical psoriasis research. Mechanistically, damaged mitochondria release oxidized mtDNA into the cytosol or extracellular space, where it engages the cGAS–STING pathway to amplify type I interferons and supportive cytokines (35, 103). In murine models, pharmacological administration of the STING antagonist H-151 effectively diminishes IMQ-induced epidermal thickening and dermal immune infiltration (36). Similarly, pharmacological inhibition of *HDAC3* reduces cellular oxidative stress and limits cytosolic mtDNA accumulation in preclinical models, thereby alleviating skin lesion severity (35). While these preclinical findings underscore the therapeutic potential of targeting nucleic acid-sensing cascades, translating these findings to human disease requires careful consideration of systemic antiviral surveillance, as broad STING inhibition may compromise physiological host defense (104).

Beyond nucleic acid sensing, excessive mtROS act as critical intracellular drivers that fuel both NLRP3 inflammasome assembly and NF- κB activation across keratinocytes and myeloid subsets. In both cultured primary keratinocytes and IMQ-induced psoriasiform mouse models, selectively scavenging mtROS with organelle-targeted antioxidants, such as Mito-TEMPO or MitoQ, blunts inflammatory signaling, suppresses keratinocyte hyperproliferation, and significantly reduces the secretion of core pathogenic cytokines, including IL-1β, IL-6, and IL-17A (105). In parallel, pharmacological activation of the Nrf2 antioxidant axis reinforces endogenous mitochondrial redox defenses in cytokine-stimulated keratinocytes and attenuates epidermal hyperplasia in psoriasiform mice (106). However, translating mtROS-directed therapies to the clinic reveals a fundamental immunometabolic dilemma governed by redox hormesis: while pathological mtROS bursts trigger oxidative damage and tissue inflammation, physiologically calibrated baseline mtROS flux acts as an indispensable signaling rheostat sustaining homeostatic signaling pathways (107, 108). Consequently, non-selective or excessive quenching of mtROS risks disrupting cellular redox equilibrium and depleting basal intracellular ROS pools, which are strictly required for physiological antibacterial host defense, autonomous cell signaling, and homeostatic epidermal differentiation and stratification (109, 110).

Furthermore, the fundamental role of mitochondrial metabolic reprogramming in orchestrating T-cell lineage activation has progressively emerged as a frontline therapeutic frontier. Pharmacological strategies aimed at rejuvenating mitochondrial OXPHOS capacity or modulating immune cell metabolic flux via mechanistic target of rapamycin (mTOR) inhibitors hold immense promise for mitigating the auto-amplifying immune responses in psoriasis. For instance, rapamycin, by selectively inhibiting the mTOR signaling node, successfully dismantles the pathological glycolytic metabolic reprogramming characteristic of Th17 cells, thereby drastically curtailing their release of downstream inflammatory mediators (111, 112).Complementing this approach, restoring the OXPHOS efficiency and mitochondrial integrity of Treg cells offers an elegant mechanism to rescue and reinforce peripheral immunological tolerance, offering a holistic framework for metabolic resetting (89, 113).

Pathological activation of DRP1 represents another pivotal therapeutic node that drives excessive mitochondrial fission, electron transport chain uncoupling, and sustained pro-inflammatory signaling across keratinocytes, macrophages, and effector T cells (7, 52, 76). In both cultured myeloid cells and psoriatic skin-induced psoriasiform mouse models, pharmacological inhibition of DRP1 using small molecules, such as Mdivi-1, restores tubular mitochondrial networks, diminishes mtROS generation, and significantly attenuates epidermal hyperplasia and cutaneous immune infiltration (52, 114). However, the clinical translation of systemic DRP1 inhibitors is constrained by a narrow therapeutic window: physiological DRP1-mediated fission and baseline mitophagy are indispensable for maintaining mitochondrial quality control in energy-demanding tissues, particularly the myocardium and central nervous system (115).

Furthermore, targeting the metabolic dependence of skin-resident memory T_RM_ cells on exogenous lipid scavenging offers an attractive potential mechanism to combat disease recurrence in psoriasis (80, 83). Foundational studies in cutaneous T_RM_ biology demonstrated that genetic ablation or pharmacological blockade of CD36 using CD36-blocking antibodies, as well as inhibition of fatty acid oxidation, effectively starves T_RM_ cells of essential energetic substrates to induce their metabolic exhaustion and loss of persistence (80). Although translating this paradigm into psoriasis therapeutics represents a compelling concept for purging pathogenic tissue-resident clones, this approach must overcome selectivity hurdles—specifically, the need for skin-confined delivery to avoid impairing protective Treg cells that likewise rely on oxidative lipid metabolism to sustain peripheral tolerance.

Consequently, non-selective systemic administration raises substantial concerns regarding off-target organ toxicities, emphasizing the imperative to develop skin-restricted topical formulations or targeted nanocarriers that selectively arrest local cutaneous fission without disrupting systemic organ homeostasis. Concurrently, as downstream conduits of severe organelle injury, mitochondria-derived extracellular vesicles (MDEVs) traffic oxidized mtDNA and lipid peroxides across the dermo-epidermal junction to prime resident immune subsets (100, 116). Disrupting this intercellular secretory and uptake circuitry—either by inhibiting keratinocyte MDEV release kinetics or blocking recipient scavenger receptor-mediated endocytosis—provides a complementary strategy to intercept localized inflammatory propagation without perturbing systemic mitochondrial dynamics (101, 102).

While modulating mitochondrial pathways presents attractive conceptual advantages, several fundamental bottlenecks impede immediate clinical translation. A primary limitation is the lack of cell-type and tissue selectivity. As summarized in **Table 1**, diverse cellular subsets within the psoriatic plaque display distinct, and sometimes reciprocal, metabolic dependencies; for instance, while pathogenic Th17 and $T_{RM}$ cells thrive on active lipid and mitochondrial metabolism, protective regulatory T (Treg) cells strictly require intact OXPHOS to sustain immunosuppression80,86. Consequently, non-targeted systemic interventions—such as systemic DRP1 inhibition, broad-spectrum ETC uncoupling, or unselective lipid uptake blockade—risk concurrently crippling host immune tolerance and causing organ toxicity (**Table 2**). Furthermore, redundant signaling pathways frequently circumvent single-metabolic-node blockades, and systemic disruption of key nodes like mTOR or STING risks immunosuppression and opportunistic infections (**Table 2**). Overcoming these translational hurdles will necessitate the development of cell-specific delivery platforms, such as topical formulations, receptor-targeted lipid nanoparticles, or spatiotemporally resolved therapeutic regimens that selectively target diseased cutaneous compartments without disrupting systemic metabolic homeostasis.

**Table 1:** Metabolic features, mitochondrial perturbations, and pathogenic implications across cutaneous and immune subsets in psoriasis.

Cell type/subset	Predominant metabolic dependency	Mitochondrial alterations & phenotypes	Underlying molecular mechanisms	Pathogenic implication	Ref
Suprabasal Keratinocytes	Aerobic glycolysis	Excessive fission, cristae fragmentation, and mtROS burst	CD147–GLUT1 activation; *HDAC3* upregulation driving MOMP and cytosolic oxidized mtDNA extrusion	Sustains epidermal hyperproliferation; activates cytosolic cGAS–STING axis to amplify localized inflammation	(33, 35, 36)
Basal Keratinocytes	Functional OXPHOS	Enhanced respiratory chain flux; membrane lipid peroxidation	*COX7B* upregulation; exhaustion of the GPX4 antioxidant axis coupled with labile iron overload	Preserves basal proliferative stemness; ferroptotic membrane rupture releases secondary DAMPs	(34, 37, 38)
M1-Polarized Macrophages	Aerobic glycolysis & *de novo* lipogenesis	Accelerated fission, loss of membrane potential, and interrupted TCA cycle	4-1BBL signaling; FABP5-mediated saturated FA uptake; GDAP1L1/DRP1 axis activation; succinate accumulation	Locks pro-inflammatory phenotype; promotes IL-1β, TNF- α, and IL-6 secretion; SUCNR1 paracrine priming	(49, 50, 52, 95)
cDCs	Glycolytic reprogramming	Decreased OXPHOS; accumulation of TCA cycle intermediates	Retrograde mtROS signaling and metabolite accumulation supporting transcriptional and epigenetic remodeling	Fulfills high bioenergetic and biosynthetic demands for antigen presentation and IL-12/IL-23 production	(53, 54)
pDCs	Functional OXPHOS & FAO	Intact respiratory chain activity and balanced redox state	Mitochondrial respiration sustains cellular ATP pools required for endosomal TLR trafficking and IRF7 translocation	Crucial for TLR7/9-mediated sensing of nucleic acid complexes to drive early massive IFN- α production	(55, 56)
Neutrophils	Glycolysis coupled with respiratory burst	Pathological hyper-fission and loss of mitochondrial membrane potential	DRP1 hyperactivation uncouples electron transport, driving an mtROS surge that triggers NLRP3 inflammasome assembly	Licenses programmatic NETosis extrusion; releases oxidized mtDNA/LL-37 complexes to sustain plaque chronicity	(61, 63, 64, 69)
Th17 & γδT17 Cells	Dual glycolysis & OXPHOS; ACC1-dependent lipogenesis	DRP1-mediated dynamic branching; sustained baseline mtROS signaling	ACC1-derived lipids act as endogenous ligands activating RORγt; mitochondrial translation supports cytokine synthesis	Fuels clonal expansion and pathogenic cytokine release (IL-17A, IL-17F, IL-22) to drive plaque development	(75, 77, 79, 80)
T_RM_ Cells	Exogenous lipid uptake & mitochondrial FAO/OXPHOS	High respiratory reserve capacity; calibrated mtROS equilibrium	CD36-mediated fatty acid scavenging fuels fatty acid β-oxidation to maintain organelle integrity	Confers long-term survival in cleared skin; acts as the primary orchestrator of localized disease relapse	(80, 83–85)
Treg Cells	Strict reliance on mitochondrial OXPHOS & FAO	Mitochondrial structural damage, cristae dilation, and impaired mitophagic clearance	Complex III dysfunction and dampened AMPK activity disrupt *FOXP3* transcriptional stability and suppressive function	Causes loss of peripheral immune tolerance; predisposes Tregs to plastic conversion into pro-inflammatory subsets	(41, 72, 86, 89, 113)

**Table 2:** Translational bottlenecks, mechanistic safety hazards, and delivery strategies for mitochondrial-targeted therapies in psoriasis.

Targeted axis/pathway	Representative compounds	Preclinical efficacy & phenotype	Mechanistic safety concerns & bottlenecks	Proposed delivery & countermeasure Strategies	Ref
DRP1-Mediated Fission	Mdivi-1, P110	Restores tubular mitochondrial networks; blunts mtROS surge; attenuates IMQ-induced epidermal hyperplasia	Narrow therapeutic window: Basal DRP1 fission is strictly required for physiological mitophagy in high-energy organs	Skin-restricted topical formulations; dissolving microneedles to avoid systemic exposure	(52, 114, 115)
mtROS & Redox Signaling	MitoQ, Mito-TEMPO	Organelle-targeted ROS scavenging; suppresses NLRP3 assembly and IL-1β/IL-17A production	Disruption of redox hormesis: Indiscriminate ROS depletion compromises physiological host defense and disrupts normal epidermal differentiation	Stage-restricted dosing limited to the acute flare phase; precise topical dose titration	(105, 107, 108, 110)
cGAS–STING Axis	H-151	Blocks cytosolic mtDNA-driven sensing; suppresses downstream type I IFNs and dermal leukocyte infiltration	Host defense impairment: Broad systemic STING blockade risks compromising physiological antiviral surveillance and antitumor immunity	Lesion-confined topical delivery; receptor-targeted nanoparticle encapsulation	(35, 36, 103, 104)
mTOR/Glycolytic Flux	Rapamycin, Everolimus	Dismantles Th17 glycolytic reprogramming; curtails pathogenic cytokine output (IL-17A/F)	Global mTOR blockade induces metabolic dysregulation, dyslipidemia, and impaired epithelial wound healing	Low-dose topical formulations; pulse dosing during acute inflammatory flares	(108, 109, 111, 112)
Fatty Acid Oxidation & CD36	CD36-blocking antibodies	Deprives T_RM_ cells of energetic fuel; induces T_RM_ metabolic exhaustion to delay psoriasiform relapse	Protective Tregs strictly depend on FAO/OXPHOS; systemic CPT1 blockade exerts hepatic steatosis risks	Confining treatment strictly to the stable/remission phase; topical epidermal lipid-transporter targeting	(80, 83, 86, 89)
MDEV Biogenesis & Secretion	GW4869, Scavenger receptor blockers	Prevents oxidized mtDNA and oxidized lipid packaging; blocks dermo-epidermal inflammatory crosstalk	Difficulty in selectively targeting MDEVs without disturbing physiological exosome trafficking	Receptor-mediated uptake inhibitors tailored to lesional macrophages; topical anti-EV decoy traps	(100–102, 116)

## 8 Conclusion and future perspectives

While the pathophysiological links of mitochondrial dynamics in psoriasis are increasingly recognized, pivotal knowledge gaps preclude immediate clinical translation. Psoriatic lesions exhibit pronounced spatiotemporal heterogeneity; yet, the precise, divergent metabolic profiles of mitochondria across distinct disease stages and highly compartmentalized cellular subclusters remain elusive. This ambiguity poses a major pharmacological dilemma: indiscriminate systemic metabolic modulation inevitably fails to reconcile the concurrent suppression of pathogenic effector T cells with the functional rejuvenation of protective Treg cells, underscoring a critical lack of cell-type-specific and context-dependent metabolic targeting strategies.

Furthermore, precise therapeutic windows for modulating mitochondrial quality control circuits, alongside targeted strategies to selectively intercept mitochondria-derived danger signals, remain incompletely defined.

In conclusion, managing psoriatic inflammation requires moving beyond broad immunosuppression toward precision, cell-specific immunometabolic regulation. Deepening our molecular understanding of these compartmentalized mitochondrial pathways will provide an actionable foundation for next-generation targeted therapeutics.
